# A Rare Case of Gastric Lipoma Presenting with Gastric Outlet Obstruction Treated Endoscopically

**DOI:** 10.1155/2019/5749830

**Published:** 2019-02-13

**Authors:** Ahmad Sharayah, Dileep C. Unnikrishnan, Akhila Arya Perumangote Vasudevan, Noor Hajjaj, Rishi Raj, Kenneth Belitsis

**Affiliations:** ^1^Internal Medicine Department, Monmouth Medical Center, Long Branch, NJ 07740, USA; ^2^Internal Medicine Department, Govt Medical Collage, Kozhikode, India; ^3^Faculty of Medicine, University of Jordan, Amman, Jordan; ^4^Gastrointestinal Department, Monmouth Medical Center, Long Branch, NJ 07740, USA

## Abstract

An 85-year-old male referred to the Gastroenterology (GI) clinic with three-month history of failure to thrive and three-week history of nausea, vomiting, and melanotic stools. Ulcerative mass obstructing gastric outlet was found on endoscopy and on follow-up CT abdomen a homogeneous submucosal mass in the gastric antrum was identified. Radiological diagnosis of giant gastric lipoma was established and patient was evaluated for surgery and, however, was rendered unfit for surgery due to his comorbid conditions. Patient was taken for endoscopic resection of the mass. On endoscopy, only partial resection was achieved due to the size of the mass, but endoloops were deployed at the stalk at the end of the procedure in hope of limiting blood supply to the lesion. On six-week follow-up endoscopy, patient's mass had completely disappeared with limited scar tissue at the site.

## 1. Introduction

Symptomatic gastric lipomas are rare neoplasms of GI tract. Bleeding due to mucosal erosions on top on the lipomas and mass effect leading to gastric outlet obstruction are some of the common presentations in such cases. Due to its rarity, there is no generalized consensus on the management. Surgical resection of the mass is the common approach in such cases, but in cases where debility or other comorbidities preclude surgery, endoscopic resection and debulking can be attempted. Here we present the case of a giant gastric lipoma where surgical resection was not feasible due to patient's comorbidities. Endoscopic resection was attempted; however due to the size and location of the lesion, only partial resection was achieved. At the end of the procedure, using a needle knife, base of the lesion was cut to create deep grooves at the stalk to prevent slipping of endoloops and an endoloop tightly encircling the stalk was left in place. On six-week follow-up, there was complete disappearance of the mass with minimal scarring of the site. Endoscopic loop placement to limit blood supply can be attempted in the management of large gastric lipoma to prevent major surgeries and its associated morbidity.

## 2. Case Presentation

**Figure 1:**
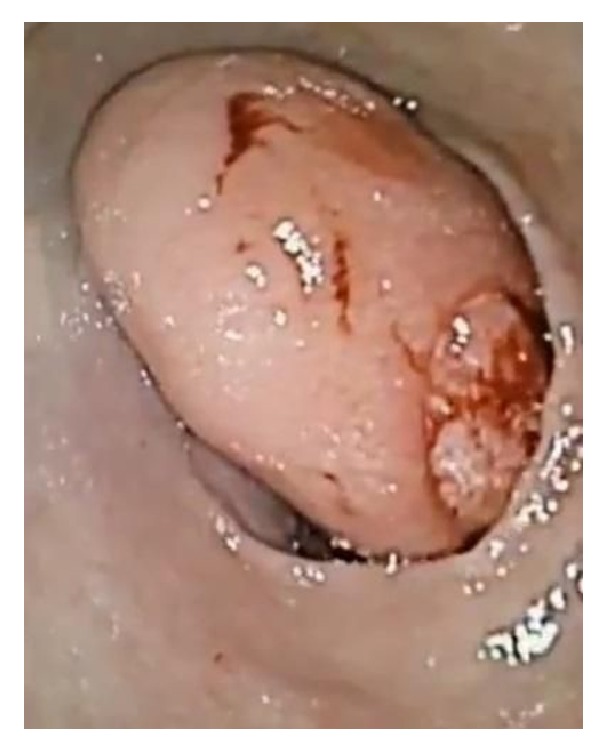
First endoscopy showing large gastric antral mass with friable ulcer at the top.

**Figure 2:**
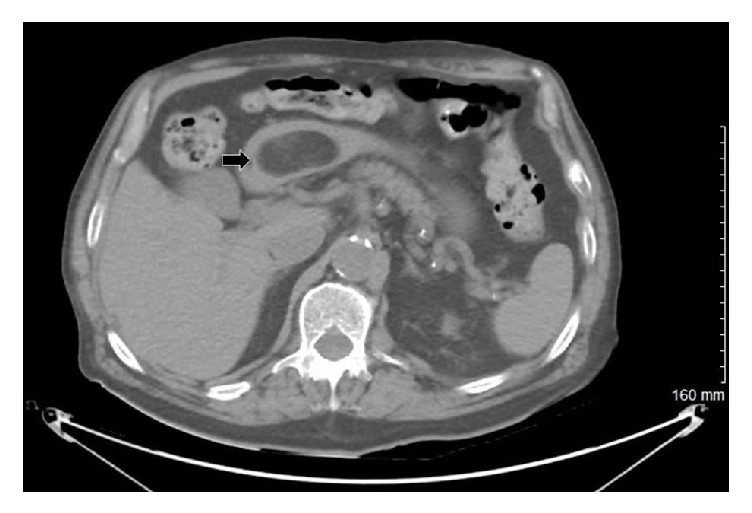
Computerized tomography abdomen. Black arrow showing gastric antral submucosal mass which is homogeneous in density and has sharp rounded borders. This is indicative of gastric lipoma.

An 85-year-old male was referred to GI clinic by his primary care physician for evaluation of anemia, weight loss, and positive stool occult blood. On obtaining a detailed history he admitted to having early satiety for the past three months and nonbilious vomiting and colicky epigastric abdominal pain for the past three weeks. His physical examination was pertinent for pallor and his abdomen was soft and nontender with no apparent swelling or hepatosplenomegaly. An esophagogastroduodenoscopy (Video 1) was performed which showed a large mass in the gastric antrum obstructing the gastric outlet with a nonbleeding but friable ulcer on top [Figure 1]. Biopsies were sent due to the concern for Gastrointestinal Stromal Tumor (GIST), gastric lymphoma, or adenocarcinoma stomach. A Computerized Tomography (CT) abdomen was ordered to further look into the etiology of the mass and determine the size and presence of lesions elsewhere. The CT abdomen [Figure 2] revealed a homogeneous submucosal mass of 5 cm x 2.5 cm size. The lesion was in the gastric antrum, homogeneous, and well contained within the gastric wall. There were no lymphadenopathy or remote lesions in the abdomen. This together with endoscopic features of the mass was suggestive of benign gastric lipoma. Surgical referral was done for possible surgical removal; however, it was advised to try to endoscopically resect or at least debulk the mass for palliation of his symptoms before attempting a surgical removal because of patient's debility and comorbidities.

**Figure 3:**
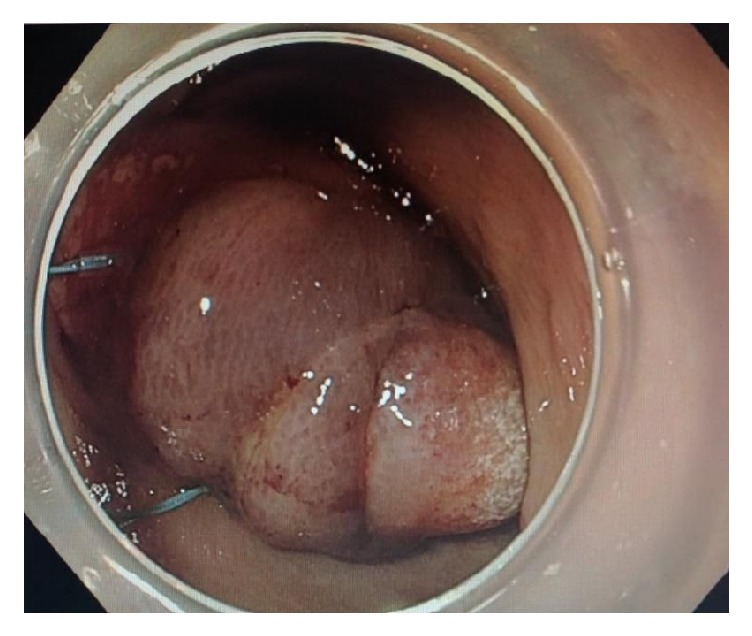
Endoscopic image of the mass after deployment of two gastric endoloops at the base.

After the failure of initial attempts to do a complete resection, partial piecemeal resection was made with the aim of debulking the lesion to relieve symptomatic gastric outlet obstruction. To limit bleeding, endoloops were deployed at the base of the lesion prior to the start of resection. Because of the location and size of the lesion, only 2 cm by 2 cm of the mass was resected. At the end of the procedure, one of the endoloops remained deployed tightly at the base protecting the margins and limiting blood supply [Figure 3]. The base of the lesion was cut using a needle knife to create shelves for the deployment of endoloops and prevent their slipping. After procedure, patient had good relief of symptoms. The pathology of the excision biopsy that came back positive for fatty tissue confirms the diagnosis of lipoma.

**Figure 4:**
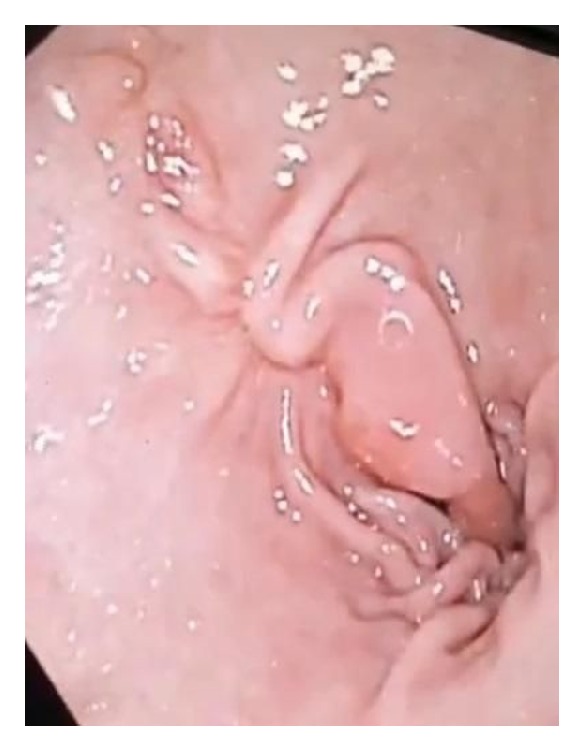
Near complete resolution of the mass with minimal scarring and residual tissue at the site on six-week follow-up endoscopy.

Repeated EGD (Video 2) at four-week interval revealed well-healed scar at the site of the lipoma without ulceration [Figure 4] and the patient at twelve-week follow-up remained asymptomatic.

## 3. Discussion

Lipomas of GI tract are rare in occurrence (1 in 600). These are the third common benign tumors in GIT after adenomas and hyperplastic polyps. These are intramucosal mesenchymal tumors of mature adipocytes and are often incidental findings on endoscopy or colonoscopy [1]. Lipomas occurring in stomach account only 1-3% of all gastric tumors. These are benign and slow growing lesions and commonly asymptomatic. When symptomatic, they can cause bleeding, abdominal pain, and intestinal intussusception. Gastric lipomas larger than four centimeter in size are more likely to be symptomatic and the common presenting symptoms are that of acute upper GI bleeding and gastric outlet obstruction.

In radiology, a well-defined and homogeneous mass in the stomach wall is suggestive of gastric lipoma. On endoscopic ultrasound, the lesion shows up as a homogeneous hypoechoic lesion. Biopsy is rarely needed for a diagnosis and endoscopic signs such as Cushing sign and tenting sign combined with EUS or CT scan are usually diagnostic.

In addition, because of the submucosal location, routine biopsies are never deep enough to secure tissue to make an accurate diagnosis [2].

Treatment for gastric lipoma is controversial. Because of its rarity, a standardized approach in treating large symptomatic lesions has not yet been established [3]. Generally asymptomatic small lipomas do not need surgical or endoscopic excision. Endoscopic polypectomy can be attempted for lipomas smaller than 3 cm; however larger broad based tumors have higher risk of perforation by endoscopic approach [4]. Surgical excision is resorted to in such cases.

Our patient, because of his cardiovascular risk factors, was not a candidate for surgery. The technique of limiting blood supply to the lesions using an endoloop resulted in complete resolution of the lesion. This technique which has already been used for other benign gastric lesions such as colonic polyps can be used effectively for cases of gastric lipoma as well. Such endoscopic approaches can be a good alternative to surgery and can prevent significant morbidity and mortality. Gastric lipomas are benign mesenchymal tumors of adipocytes commonly seen in the gastric antrum. They are diagnosed by the homogeneous appearance, well-defined borders, and submucosal location in imaging studies. In the rare situations where they cause symptoms, they can cause symptoms of upper gastrointestinal bleed or gastric outlet obstruction. Symptomatic gastric lipoma is a rare occurrence. Because of the rarity, there is no consensus on the treatment in such cases. Majority of cases are managed through surgical resection which can cause significant morbidity. In a systematic review of 32 cases of large gastric lipoma published by Cappell et al. [5] the vast majority of cases still opted for surgical resection of the tumor to endoscopic therapy. The various procedures performed in these cases included submucosal mass excision and enucleation of the lesion. Partial and in rare cases total gastrectomy accompanied these procedures depending on the degree of wall involvement by the tumor. In minority of cases, endoscopic and laparoscopic transgastric resection were performed. These methods cause less morbidity to the patient if successful, however has the risk of causing gastric perforation. Limiting blood supply to the mass using an endoloop applied at the base may lead to spontaneous resolution of the lesion as it was observed in our case. This can be attempted safely to avoid the morbidity of a surgery.

## Supplementary Materials

Supplementary 1 Video 1: first endoscopy which shows a large gastric antral mass with friable ulcer at the top. The movement of mass with peristalsis in and out to the duodenum through the gastric outlet can be appreciated, which is leading to the gastric outlet obstruction.

Supplementary 2 Video 2: postprocedure endoscopy at six-week follow-up. There is near complete resolution of the mass with minimal scarring and residual tissue at the site.
